# Western gray whales and seismic operations: an introduction to the topical selection and a tribute to the late Rodger Melton

**DOI:** 10.1007/s10661-022-10056-1

**Published:** 2022-10-18

**Authors:** Bill Streever

**Affiliations:** S/V Rocinante, 3824 Cedar Springs Rd 801-2771, Dallas, TX USA

For some time, people have known that baleen whales respond to underwater sounds. Take for example Arthur Conan Doyle; in 1880, long before creating the character Sherlock Holmes, Doyle sailed to the Arctic and commented in his journal about whales reacting to sounds from the newly introduced steam-powered whalers (Doyle, 2012). Or take as another example from the same era, Captain Foley of the whale ship *Monterey,* who wrote of bowhead whales distinguishing between the natural sounds of floating ice and the sounds of boats gently bumping against that ice. Natural sounds elicited no apparent reaction, while whales scattered in reaction to the sounds of boats bumping floes (Bockstoce, 1986).

But the knowledge that whales respond to sounds is nothing more than a starting point. A far more important question revolves around knowing how different kinds of whales are impacted by sounds in different situations. The distinction is important. In this world crowded with humans and overrun by human activities, there is a need to understand how sounds might or might not affect the health of both individual whales and whale populations. Will behavioral responses to anthropogenic sounds move whales away from food resources, away from mates, away from offspring? Or are the behavioral responses benign? Or, as seems increasingly likely, do responses vary from harmful to benign depending on circumstances?

Such questions pose immense challenges. To begin, take the sound itself. Before any serious thought can be given to behavioral impacts, the sound itself has to be understood. Is it impulsive or continuous? Is it directional? Does it rise suddenly and without warning? What frequencies make up the sound?

Next, how does the sound travel through water? The degree to which a sound diminishes with distance from the source varies with everything from its frequency composition to water depth to bottom characteristics to water temperature, all of which vary across locations and across a sound’s travel path.

Add to these complexities the issue of background sounds. Are anthropogenic sounds potentially less problematic in inherently noisy environments than they would be in quieter realms? For example, are anthropogenic sounds less relevant in the wave battered shallows frequented by the feeding western gray whales (*Eschrichtius robustus*) that are the subject of this special issue than they would be for whales in deeper, quieter waters?

Also, consider a whale that hears a sound. Testing hearing in humans is simple. Put a person in a sound-proof room, play tones of different frequencies and volumes, and ask for a response each time a sound is heard. The result is an audiogram, a plot of hearing sensitivity against frequency. The same can be done with trained animals. It has in fact been done with various seals, some toothed whales, and even polar bears (e.g., Bowles et al., 2008; Kastelein et al., 2003, 2009). But baleen whales are animals that cannot be kept under controlled conditions, and while it seems likely that they could, in principle, be trained, the devil of such training may lie in the easily imagined details. And so their hearing abilities have to be surmised through other means.

Move on now to the next step, that of associating a received sound with a change in behavior. Do baleen whales, hearing a sound, change course (e.g., Malme et al., 1988)? Does their calling behavior change (e.g., Blackwell et al., 2015)? Does their distribution change (e.g., MacDonald et al., 2012; Yazvenko et al., 2007a)? Does their diving behavior change (e.g., Friedlaender et al., 2016)? While challenging logistically, in many ways this step is straightforward. All that is required is a method of following whales as they are exposed to sounds.

But how do we determine if well-documented changes in behavior are relevant to the survival of individuals and the well being of populations? Baleen whales are, for the most part, long distance migrators. If in responding to a sound a whale moves a few kilometers out of what appeared to be its intended path, does it suffer? What if it moves from an area of high prey density to an area of low prey density? What if it changes its diving behavior in a manner that renders feeding ineffective? 
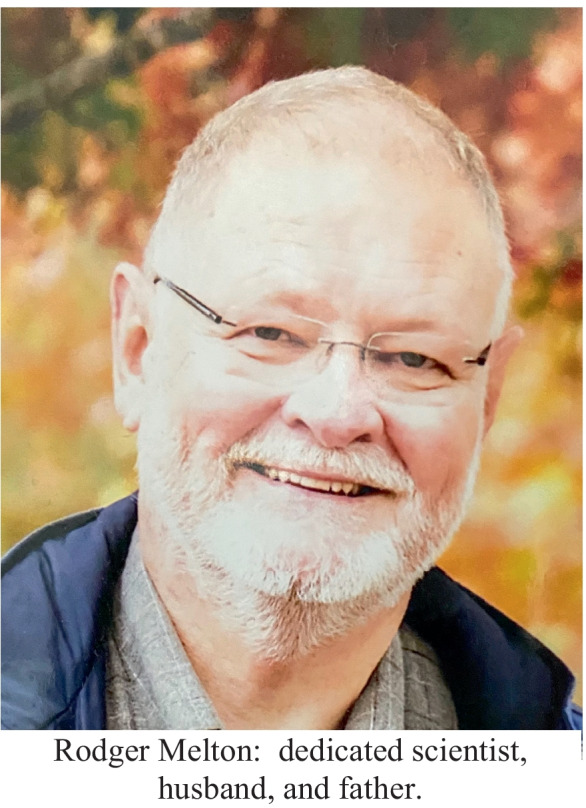


With all of that by way of background, it is time to introduce an exceptional man, Huel “Rodger” Melton. Rodger, with a Ph.D. in chemical engineering, spent a career working for ExxonMobil. During that career, he became the company’s Chief Environmental Scientist, a job that not only allowed but also required him to provide advice on difficult topics, to interface between a major oil and gas company on the one hand and the environmental community on the other, and to initiate and organize research on potentially contentious issues in a world where environmental risks and impacts require urgent attention.

In 2001, Rodger became aware of his employer’s plans to undertake seismic operations near Piltun Bay, Sakhalin Island, in far eastern Russia. Seismic operations use powerful sound sources, called air gun arrays, to image subsurface geology. A number of marine mammal species use the shallow waters near Piltun Bay. Among these is the western gray whale, thought to be extinct until the late 1970s (Blokhin et al., 1985; Brownell & Chun, 1977) and in 2001 considered to be critically endangered. The shallow waters near Piltun Bay are the summer feeding grounds of the western gray whale. The possibility that seismic operations might harm gray whales could not be denied. Controversy was inevitable.

At that time, Rodger would not have called himself a marine mammal specialist. In fact, I suspect that he never considered himself to be a marine mammal expert even years later, despite his intense involvement in the field for well over a decade. But he did—and very much correctly so—see himself as a scientist. And as a scientist, he leapt into the potentially troubled waters near Piltun Bay. He read voraciously, absorbing the content of hundreds of papers and books in the fields of underwater acoustics, marine mammalogy, and behavioral ecology. He talked to academics, government scientists, and consultants around the world, always searching for clarification. He worked late nights and early mornings, both fascinated by and passionate about the issues at hand.

And almost immediately, he realized four things. First, ExxonMobil would have to develop its own research program, one grounded in the traditions of the scientific method and peer review, and that did not reject reasonable collaborative opportunities, yet one that could stay focused on the company’s practical needs. Second, that program would have to integrate oil and gas industry operational requirements with monitoring, mitigation, and data collection. Third, the program would require a multidisciplinary and multinational collaboration that teamed Russian scientists with internationally renowned non-Russian experts. And fourth, he realized, perhaps as early as 2001, that the question of interest was not “do gray whales respond to underwater sounds?” but rather “do gray whale responses have the capacity to harm individuals or, even worse, to harm the entire population?” By around 2010, Rodger was casually referring to this last question as “the what it all means” question, the key conservation driver behind concerns about underwater sounds and marine mammals.

The work done under Rodger’s leadership in 2001—in his early years as a whale researcher—resulted in a collection of papers published in *Environmental Monitoring and Assessment* in 2007. Two of these papers, one listing Rodger as a coauthor, plainly recognized that gray whales responded to air gun sounds. In one, the authors documented significant correlations “between five measures of western gray whale movement and behavior patterns and various measures of potential impact from the marine 3-D seismic survey” (Gailey et al., 2007). In the other, authors reported additional statistically significant behavioral responses and changes in gray whale distribution, but also noted that “gray whales remained in the Piltun feeding area” throughout the operations and that “the total numbers of gray whales observed in the Piltun feeding area were approximately constant during the pre-seismic and seismic survey periods” (Yazvenko et al., 2007a). In another paper from the same special issue, the authors, including Rodger, concluded that “the 2001 seismic survey had no measurable effect (*α* = 0.05) on bottom feeding activity of western gray whales off Sakhalin Island” (Yazvenko et al., 2007b).

At this point, it would have been a simple thing for Rodger and his colleagues to have claimed that no further research was needed. Although behavioral responses were clearly documented, the whales stayed on the feeding grounds and seemed to feed normally. It appeared that seismic operations were not going to lead to the demise of the western gray whale. But Rodger, as a scientist, knew that the work had only scratched the surface. He understood that there was more to learn.

Rodger convinced ExxonMobil to stay engaged in annual monitoring and research, some of it undertaken with other industry partners and all of it involving discussions and collaboration with Russian and non-Russian academics and government scientists as well as environmental organizations. In the ensuing years, the importance of determining the ecological and population consequences of behavioral responses to sound grew more widely discussed. The conversation was spurred on in part by the creation of what became known as the PCAD Model, for “Population Consequences of Acoustic Disturbance” (NRC, 2005), which was later modified to encompass non-acoustic disturbances to become known as the PCOD Model, for “Population Consequences of Disturbance” (New et al., 2014).

After years of monitoring, studies, and speculations about western gray whale responses to sounds, a new opportunity arose for a second round of intensive research related to seismic surveys planned for 2015. Rodger and his team of scientists, involved with program planning from the outset, devised a far more intensive sampling regime than that of 2001, and one that relied on the principles of the PCOD models.

During the planning stages of that work, Rodger was fighting an incurable form of cancer. By 2015, he was physically unable to join his colleagues and friends in the field, but his mind remained intact, robust, energetic, and engaged. The work went ahead, with Rodger often connected to eastern Russia by telephone from his home in Houston. And, or so it seemed, when he was not on the telephone, he was on email, always asking questions, raising issues, probing, conferring, and thinking.

The cancer took Rodger’s life in 2017, but the work that he set in motion continued. It was a wonderfully complex effort that coordinated mitigation and monitoring with research, that looked realistically at the tough technical issues associated with understanding a sound field as it changed over time and space, that accepted the reality of sounds coming from more than one source, and that recognized the possibility of food resources influencing whale responses to sounds.

Taken individually, many aspects of the work appeared to be of limited value. But considered collectively, as intended, the true value of the work emerged. With that in mind, it was clear that most of the papers growing from the 2015 research and monitoring effort were so interrelated that they had to be published together, as a collection.

Rodger would almost certainly agree with me that this work, as painstaking as it was, will not come close to providing definitive answers to his “what it all means” question. In fact, he might agree with me that definitive answers to such a question will remain perpetually elusive. In this as in many important questions, the issue is one of adding to the knowledge base, of providing information that can be used in future decision-making processes as humans continue to interact with their environment, and, in this case, as humans continue to interact with some of the most awe-inspiring animals in the sea: baleen whales.

Although I was not directly involved with the work described in this special issue, I was pleasantly surprised to be invited to act as its guest editor. I was pleased in part because of the topic, one I have dabbled in over the years. But I was also pleased because I knew Rodger and worked with him on several projects undertaken as part of a joint industry program. I knew him as a man of strong convictions and a strong work ethic. More importantly, I knew him as a man of great intellect, integrity, and determination. He was, in short, a person I admired, and it is my hope that this collection of papers will honor his life and his name.
